# Spread of a *Gammabaculovirus* within Larval Populations of Its Natural Balsam Fir Sawfly (*Neodiprion abietis*) Host Following Its Aerial Application

**DOI:** 10.3390/insects3040912

**Published:** 2012-09-28

**Authors:** Roger Graves, Christopher J. Lucarotti, Dan T. Quiring

**Affiliations:** 1 Faculty of Forestry and Environmental Management, The University of New Brunswick, Fredericton, New Brunswick E3B 5A3, Canada; E-Mails: rogergraves19@gmail.com (R.G.); quiring@unb.ca (D.T.Q.); 2 Natural Resources Canada, Canadian Forest Service—Atlantic Forestry Centre, 1350 Regent Street, Fredericton, New Brunswick E3C 2G6, Canada

**Keywords:** *Neodiprion abietis*, balsam fir sawfly, *Abies balsamea*, *Gammabaculovirus*, nucleopolyhedrovirus, NPV, disease transmission, pest management

## Abstract

Field trials and assessments of the balsam fir sawfly (*Neodiprion abietis*) nucleopolyhedrovirus (NeabNPV: *Baculoviridae*, *Gammabaculovirus*) against its natural host were conducted in July and August 2002 near Corner Brook, Newfoundland and Labrador, Canada, in naturally regenerated, precommercially thinned stands dominated by balsam fir (*Abies balsamea*). Two experimental blocks, each with its own untreated control, were established. The purpose of the Island Pond block was to examine the spread of NeabNPV from a 313-ha aerial treatment block out into adjacent populations of balsam fir sawflies. The purpose of the Old Man’s Pond block (2,093 ha) was to determine whether NeabNPV could disperse into populations of balsam fir sawflies within a 200-m zone between spray swaths. NeabNPV was applied to treatment blocks by a Cessna 188B AgTruck aircraft equipped with MicronAir AU4000 rotary atomizers at an application rate equivalent to 1 × 10^9^ NeabNPV occlusion bodies/ha in 2.5 L of 20% aqueous molasses. At Island Pond, NeabNPV infection increased with time following the spray, especially for individuals close to the treatment block, and infection rate decreased to a measured distance of 400 m from the treatment block. At Old Man’s Pond, NeabNPV infection rose higher (80% *vs.* 15%) and sawfly densities declined more (84% *vs.* 60%) in the area between spray swaths than in the control block.

## 1. Introduction

Development of microbial pathogens as viable alternatives to chemical pesticides has been encouraged by public pressure for environmentally benign pest management strategies. Baculoviruses are generally considered ideal candidates for biological control agents because they are naturally occurring and host-specific infecting only a single species or a few closely related species of insects, can cause epizootics in host-insect populations, and persist in the environment for many years [1,2,3,4,5]. Baculoviruses are covalently closed, double-stranded DNA viruses between 85 and 180 kb in size [6]. Currently, the family *Baculoviridae* is divided into four genera: *Alphabaculovirus* (nucleopolyhedrovirus or NPV) and *Betabaculovirus* (granulovirus or GV), both of which occur in Lepidoptera; *Deltabaculovirus*, which are NPVs in Diptera; and *Gammabaculovirus*, which are NPVs in sawflies [7]. Many studies have been conducted to assess the effects of NPVs on insect populations (e.g., [8,9,10,11,12,13,14,15,16,17,18]), with most reporting that NPV-induced mortality acts in a density-dependent manner [19] to reduce insect population densities.

The balsam fir sawfly (*Neodiprion abietis* Harris) is an eruptive defoliator that is native to North America [20,21]. In Newfoundland, Canada, adult female balsam fir sawflies oviposit eggs into slits they cut in current-year balsam fir (*Abies balsamea* (L.) Mill.) needles in September and October [22]. Eggs over-winter there, and larvae emerge in late June to mid-July the following year. Early instar balsam fir sawfly larvae are gregarious [23], and all larval instars feed on balsam fir foliage that is 1 year old and older [24,25]. Male larvae develop through five instars, over approximately 30 d, whereas females undergo five or six instars and complete larval development in about 35 d [22]. Larvae spin cocoons and pupate on the needles of balsam fir trees and emerge as adults in late August and early September. Although periodic outbreaks of balsam fir sawflies usually last 3–4 y, the length and severity of the most recent outbreak in western Newfoundland are unprecedented and have led to extensive efforts to manage balsam fir sawfly populations and minimize their impact on the region’s most important forest resource, balsam fir [18,26]. Historically, collapses of balsam fir sawfly populations have been associated with epizootics of the *Gammabaculovirus*, NeabNPV [18,22]; recent studies have shown that populations of balsam fir sawfly larvae can be successfully suppressed by aerial applications of NeabNPV [17,26,27]. Although aerial applications are generally considered to be the most efficient way to disseminate most forest and agricultural pest control products to large areas, they are often expensive to conduct. Additionally, NPVs are relatively expensive compared with other microbial control products, such as *Bacillus thuringiensis*, because commercial NPV production must be done *in vivo* [27].

Aerial field trials with NeabNPV were carried out to determine whether aerosol drift and horizontal transmission of NeabNPV in balsam fir sawfly larvae would allow reduced spray coverage to initiate NeabNPV epizootics in epidemic populations of *N. abietis*. Two experimental blocks, each with its own untreated control, were established. The first experimental block examined the spread of NeabNPV from a 313-ha aerial treatment block out into adjacent populations of naturally occurring *N. abietis*. The second experimental block (2,093 ha) was assessed to determine whether NeabNPV could disperse into populations of balsam fir sawfly larvae within a 200-m untreated zone located between two, 200-m spray swaths.

## 2. Results and Discussion

### 2.1. Island Pond

**Figure 1:**
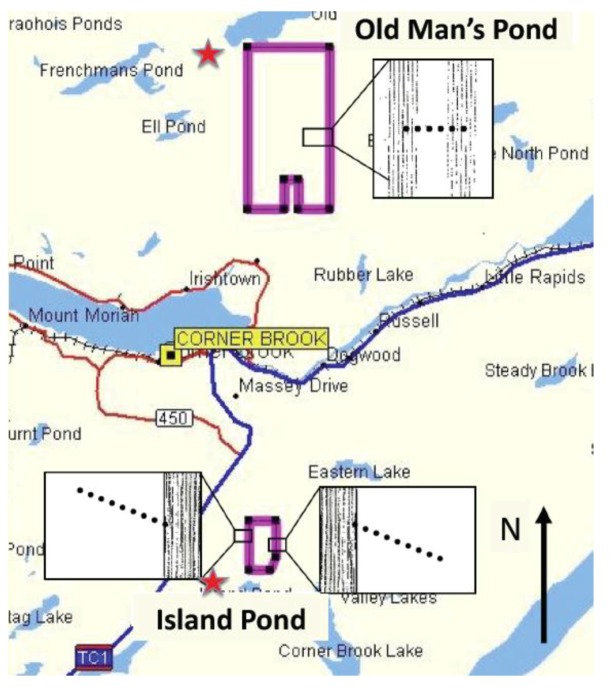
Map of area surrounding Corner Brook, NL, showing the location of Island Pond and Old Man’s Pond NeabNPV-treatment blocks in 2002. Insets show the locations of sampling transect lines (dotted lines) and aircraft spray tracts (dashed lines) at each location. Control blocks, which were located 1–5 km from treatment blocks, are indicated with stars.

**Figure 2:**
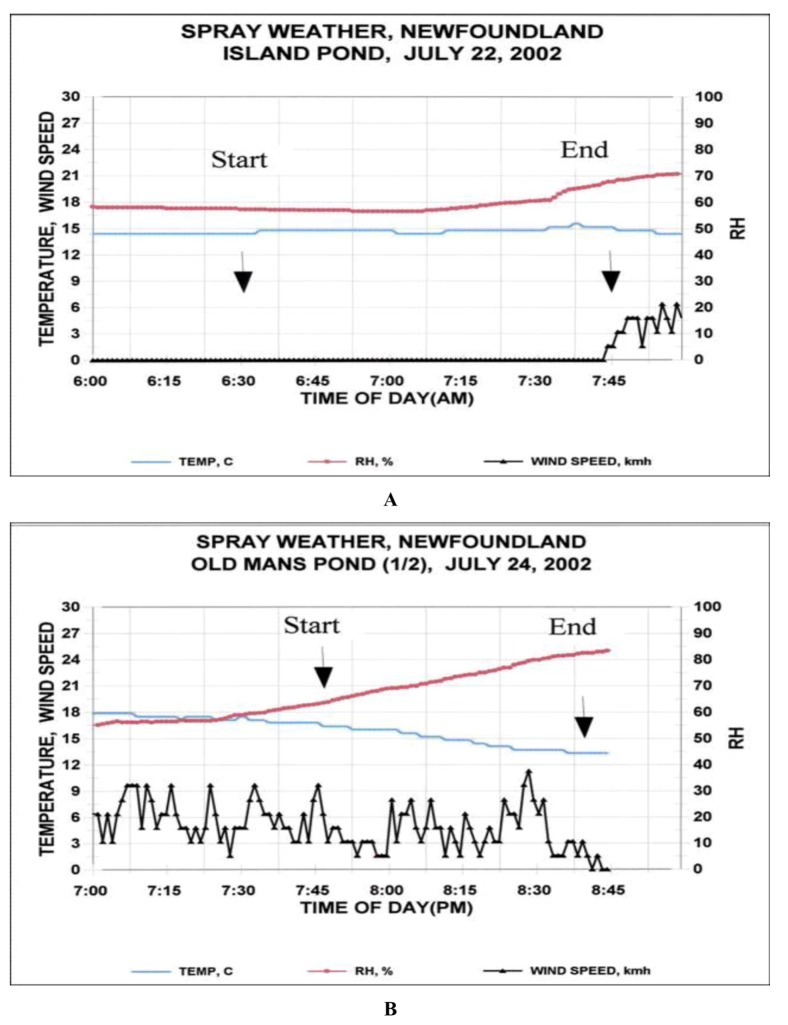
Wind speed, temperature, and relative humidity during the periods of NeabNPV aerial spray operations on 22 July 2002, at (**A**) Island Pond and on 24 July 2002, at (**B**) Old Man’s Pond.

**Figure 3:**
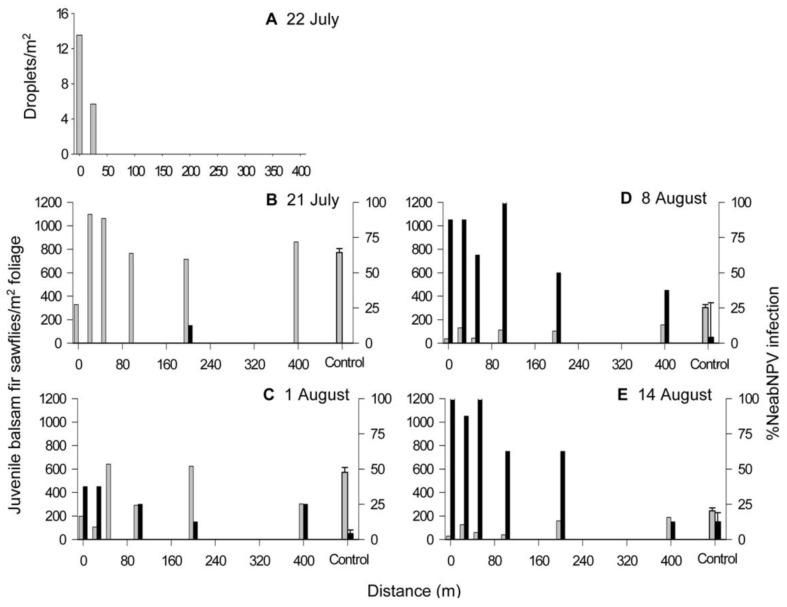
(**A**) Mean spray deposit along two transect lines extending 400 m out from the east and west sides of a 313-ha treatment block at Island Pond following aerial application of NeabNPV on 22 July 2002. Juvenile balsam fir sawfly (larvae and pupae) density (gray bars) and mean prevalence of NeabNPV infection (black bars) in the treatment block and the untreated control block on (**B**) 21 July, (**C**) 1 August, (**D**) 8 August, and (**E**) 14 August 2002. The densities and infection in the control plots are represented as a mean value (±SEM) because they exhibited no discernable consistent pattern along the transects.

During the period of the aerial application at Island Pond (Figure 1), the temperature was 14–15 °C, relative humidity increased from about 60 to 70% and wind speed was negligible at ≤1.5 km/h (Figure 2A). Spray droplets were not detected on Kromekote™ deposit cards beyond 25 m outside the treatment block (Figure 3A), on either side of the treatment block, presumably due to low wind velocities at the time of application. Also, before application of NeabNPV, densities of juvenile sawflies were relatively constant along the 400-m transect lines extending out from either side of both the treatment (Figure 3B) and control (data not shown) blocks. Consequently, data from the two respective transects in either the control or treatment blocks were pooled for analysis.

**Figure 4:**
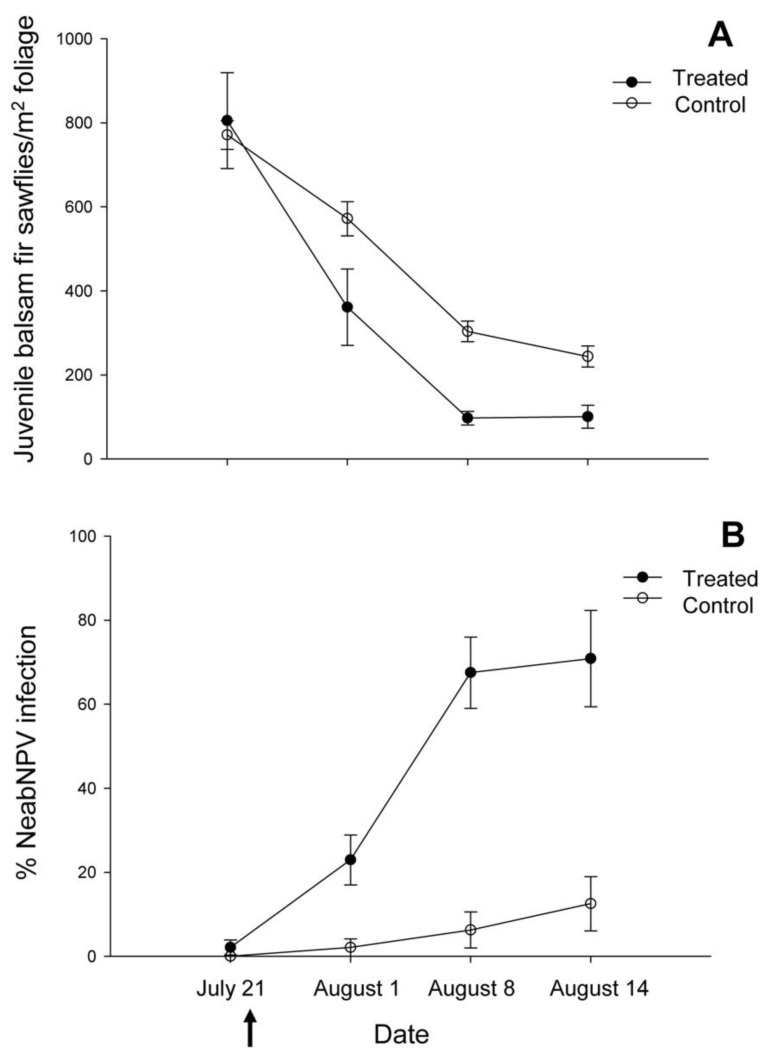
(**A**) Seasonal trends in the mean (±SE) densities of juvenile (larvae and pupae) balsam fir sawflies and (**B**) mean (±SE) prevalence of NeabNPV infection in juvenile sawfly populations adjacent to NeabNPV treatment (black circles) and untreated control (white circles) blocks at Island Pond. Arrow indicates aerial application of NeabNPV on 22 July 2002.

NeabNPV was not detected in any larvae in the control block on the day before the aerial application of NeabNPV. Densities of balsam fir sawfly larvae and/or pupae (juvenile balsam fir sawflies, henceforth) at the control block decreased with time (F_3,80_ = 34.64, *p* < 0.01) from about 770 individuals/m^2^ before the spray to fewer than 250 individuals/m^2^ 3 w after the spray (Figure 3E and Figure 4A). Densities of juvenile balsam fir sawflies were not significantly affected by distance from the hypothetical edge of the control block (F_1,80_ = 0.77, *p* = 0.579), nor were interactions between time and distance (F_3,80_ = 0.81, *p* = 0.66), or among time, distance, and treatment (*i.e.*, no treatment) (F_(3,80)_ = 0.81, *p* = 0.67) significant.

NeabNPV was only detected in three larvae, located 200 m from the treatment block, on the day before the aerial application of NeabNPV. NeabNPV prevalence was negatively correlated with distance from the treatment block in samples taken after the spray (Table 1). During the weeks following the spray, the percentage of infection with NeabNPV increased with time (Figure 3B–D and Figure 4B) (F_(3,80)_ = 41.16, *p* < 0.01). The prevalence of NeabNPV in juvenile sawflies outside the treated block rose from <5% before the spray to approximately 70% after 3 w. There was a greater increase in the prevalence of NeabNPV in juvenile sawflies at the edge of the treatment block than at the hypothetical edge of the control block (F_1,80_ = 192.90, *p* < 0.01). NeabNPV prevalence decreased with distance from the treated block (F_1,80_ = 12.93, *p* < 0.01), resulting in significant interactions between treatment and time since application of NeabNPV (F_3,80_ = 42.51, *p* < 0.01) and between treatment and distance from the treatment block (F_1,80_ = 5.64, *p* < 0.01). Similarly, the increase in NeabNPV infection along the transect outward from the treatment block resulted in a significant interaction between time and distance from the treatment block (F_3,80_ = 6.74, *p* < 0.01) and by the three-way interaction among treatment, time, and distance from the treatment block (F_3,80_ = 2.34, *p* < 0.01). By the third week after NeabNPV application, density was positively correlated with distance from the treatment block (Table 1).

**Table 1:** Island Pond 2002. Summary of Pearson correlation analyses showing weekly trends in the relationship between distance from the treated (NeabNPV) or untreated (control) blocks and juvenile sawfly densities and the prevalence of NeabNPV infection in juvenile balsam fir sawfly populations.

Treatment	Weeks post spray	N	Density Pearson’s *r*	*p* value	Infection Pearson’s *r*	*p* value
Control	1	12	−0.469	0.12	−0.343	0.27
Control	2	12	−0.254	0.43	−0.229	0.47
Control	3	12	−0.434	0.16	−0.421	0.17
NeabNPV	1	12	0.156	0.63	−0.103	0.75
NeabNPV	2	12	0.466	0.13	−0.718	0.01
NeabNPV	3	12	0.722	0.01	−0.909	0.00

### 2.2. Old Man’s Pond

**Figure 5:**
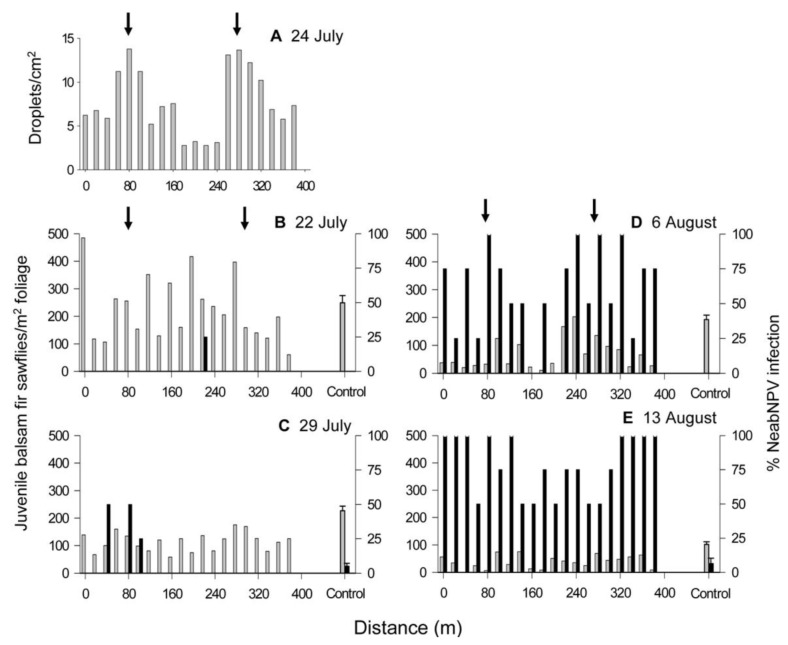
(**A**) Mean spray deposit along a transect line extending 400 m across two 50-m NeabNPV spray swaths (arrows) and a 200-m untreated zone (between arrows) at Old Man’s Pond following aerial application of NeabNPV on 24 July 2002. Juvenile balsam fir sawfly (larvae and pupae) densities (gray bars) and prevalence of NeabNPV infection (black bars) in the treatment block [same transect line as (**A**)] and the untreated control block on (**B**) 22 July, (**C**) 29 July, (**D**) 6 August, and (**E**) 13 August 2002. The densities and infection in the control plots are represented as a mean value (±SEM) because they exhibited no discernable consistent pattern along the transects.

**Figure 6:**
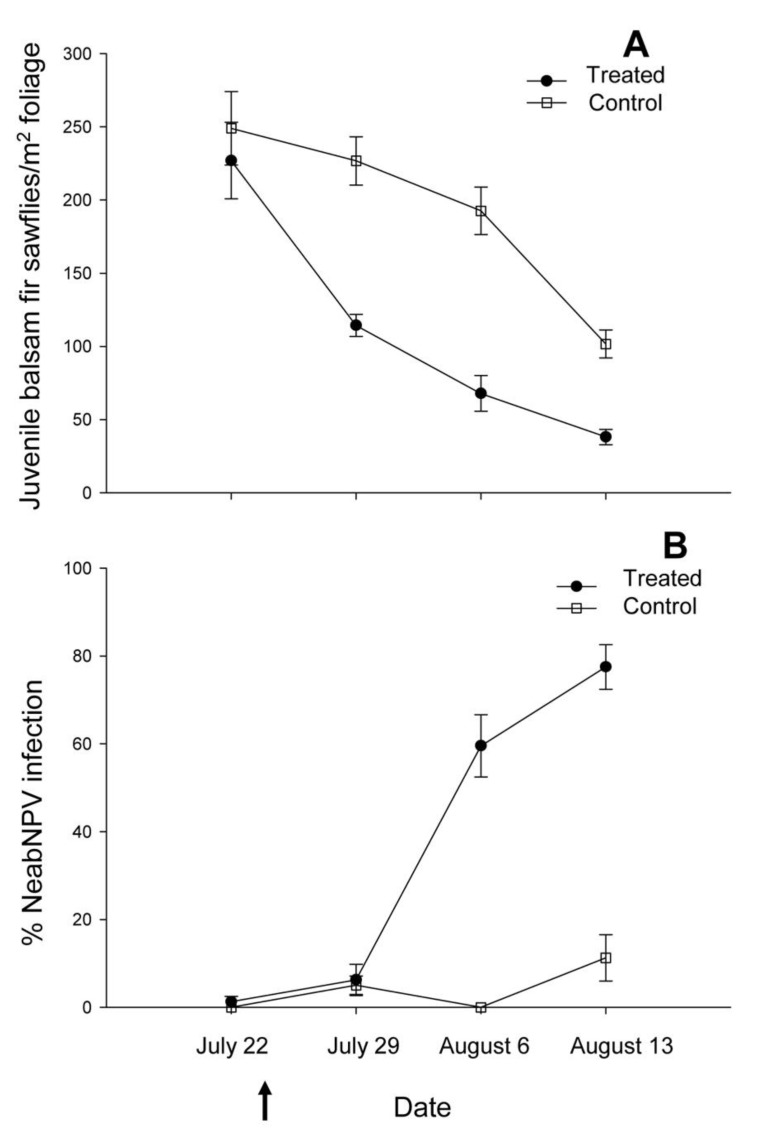
(**A**) Seasonal trends in the mean (±SE) densities of juvenile (larvae and pupae) balsam fir sawflies and (**B**) mean (±SE) prevalence of NeabNPV infection in juvenile sawfly populations adjacent to NeabNPV treatment (black circles) and untreated control (white squares) blocks at Old Man’s Pond. Arrow indicates aerial application of NeabNPV on 24 July 2002.

During the 50 min over which NeabNPV occlusion body (OB) applications occurred at Old Man’s Pond (Figure 1), the temperature declined from 16.5 °C to 13.5 °C and the relative humidity rose from 65% to 82% (Figure 2B). Wind speed was variable, gusting between 1.5 and 11.5 km/h and blew predominantly from the northwest. The exact position of the aircraft over the transect line could not be determined, but the greatest concentration of droplets occurred at points 80 and 280 m along the transect line and tapered off either side of those points (Figure 5A). NeabNPV was not detected in any larvae in the control block and was only detected in two larvae within the treatment block 2 d before the aerial virus application. Also, at this time, there was no significant correlation between larval sawfly density and distance from the spray swaths in the treatment block. As was the case at Island Pond (Figure 3B–E and Figure 4A,B), the densities of juvenile sawflies and NeabNPV infection in the control plots showed no discernable pattern along the transects, so data were pooled and are represented as mean values (±SEM) (Figure 5B–E and Figure 6A,B).

The prevalence of NeabNPV infection increased with time following the application of NeabNPV OBs (F_3,144_ = 43.20, *p* < 0.01), with 100% NeabNPV infection in juvenile sawflies at most sampling points beneath the spray swaths 3 w after the spray (Figure 5E). Overall, NeabNPV infection was much higher in the treatment block than in the control block (F_1,144_ = 97.42, *p* < 0.01). Prevalence of NeabNPV infection rose on average to 50%–75% in juvenile sawflies in the untreated zone between the spray swaths, resulting in a significant effect of distance from the spray swaths (F_1,144_ = 9.58, *p* < 0.01). This was presumably a result of aerosol spread of NeabNPV by gusting winds during the application of NeabNPV OBs (Figure 2B). The increase in prevalence of NeabNPV infection in the area between spray swaths occurred more slowly, and to a lower extent than that at sample points beneath the spray swaths, resulting in interactions between time and distance from the spray swaths (F_3,144_ = 4.22, *p* < 0.01), treatment and time since treatment (F_3,144_ = 23.53, *p* < 0.01), and treatment and distance (F_1,144_ = 7.04, *p* = 0.01). Percentage infection within the treatment block was not influenced by interactions among treatment, time, and distance from the spray swaths (F_3,144_ = 1.48, *p* = 0.22).

**Figure 7:**
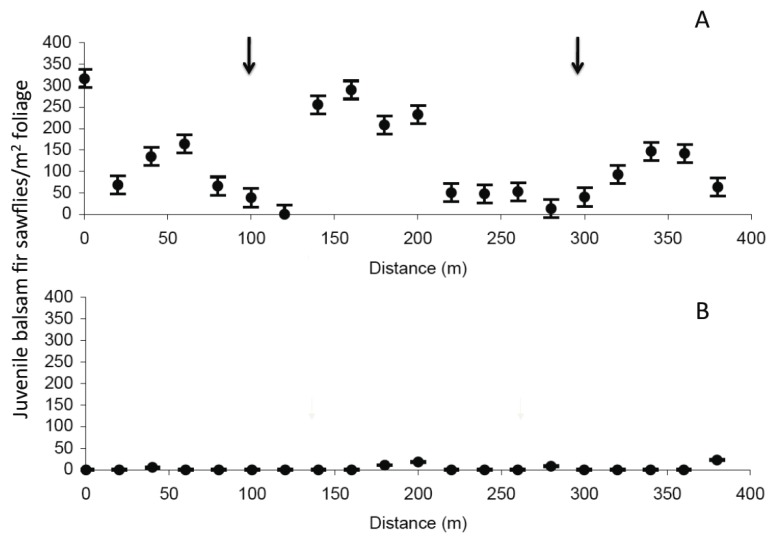
Densities of juvenile balsam fir sawflies at Old Man’s Pond on (**A**) 2 July and (**B**) 13 August 2003, 1 y after the aerial application of NeabNPV. The densities are represented as mean values (±SEM). Arrows indicate the approximate location of the original spray swaths.

Densities of juvenile sawflies varied between approximately 100 and 500/m^2^ just before the application of NeabNPV (Figure 5B and Figure 6A). Thereafter, densities of juvenile sawflies decreased with time (F_3,144_ = 12.27, *p* < 0.01) from an average of approximately 250/m^2^ before the spray to fewer than 100/m^2^ 3 w later (Figure 5B–E and Figure 6A). Juvenile balsam fir sawfly densities declined more in the treatment block than in the control block (Figure 5B–E and Figure 6A) (F_1,144_ = 12.38, *p* < 0.01). Juvenile sawfly densities were influenced by distance from the spray swaths (F_1,144_ = 4.20, *p* = 0.04), declining more at sample points within or adjacent to the spray swaths than in those toward the middle of the untreated zone. Declines in juvenile sawfly densities in the untreated zone in the weeks following the spray resulted in an interaction between time and distance from the spray swaths (F_1,144_ = 4.00, *p* = 0.01) but not between treatment and distance (F_1,144_ = 0.33, *p* = 0.57). The overall decline in juvenile sawfly densities in the treatment block in the weeks following the spray was higher than in the control block (Figure 5B–E and Figure 6A) (F_1,144_ = 12.38, *p* < 0.01), resulting in an interaction between time and treatment (F_3,144_ = 3.01, *p* = 0.03). Juvenile sawfly densities were not influenced by the interaction among time, treatment, and distance from the spray swaths (F_3,144_ = 0.24, *p* = 0.87) (Table 2). In 2003, juvenile balsam fir sawfly densities were lowest in the areas underneath the spray swaths on 2 July and were virtually zero along the entire transect by 13 August (Figure 7). Although application of NeabNPV at the rates used here does not provide foliar protection in the year of application, it can significantly reduce populations of balsam fir sawflies in the next and subsequent generations [17].

**Table 2:** Old Man’s Pond 2002. Summary of Pearson correlation analyses showing weekly trends in the relationship between distance from the two spray swaths separated by 200 m in the treatment block and the untreated (control) block and, juvenile balsam fir sawfly densities and the prevalence of NeabNPV infection in juvenile balsam fir sawfly populations. The “distance from spray” values used for the treatment block were also used for the control block, assuming that spray swaths would have occurred in the same location as in the treatment.

Treatment	Weeks post spray	N	Density Pearson’s *r*	*p* value	Infection Pearson’s *r*	*p* value
Control	1	20	−0.237	0.31	0.150	0.02
Control	2	20	0.505	0.62	-*	-*
Control	3	20	0.058	0.81	−0.408	0.07
NeabNPV	1	20	−0.418	0.07	−0.402	0.01
NeabNPV	2	20	−0.402	0.50	−0.364	0.12
NeabNPV	3	20	−0.103	0.01	−0.098	0.68

* Infection values were 0 on the second sampling date, 29 July 2002.

Application rates for NeabNPV were initially determined from published literature [3,12,20]. NeabNPV was applied at rates between 1 and 3 × 10^9^ NeabNPV OBs/ha [17], which was lower than rates used for other gammabaculoviruses against their respective sawfly hosts (reviewed by Wallace and Cunningham [20]). The lower application rates for NeabNPV were selected as a compromise between the ability to produce NeabNPV OBs in the field [27] and the large areas of balsam fir sawfly-infested forests to be treated [28,29]. These lower rates were shown to be effective for suppressing increasing and peaking populations of balsam fir sawflies in the year following application [17], where efficacy relies on infection of a few individuals within groups of sawfly larvae, followed by the production and horizontal transmission of NeabNPV to other individuals within those groups [30]. Additionally, in our experiments, we have attempted to use spray drift to our advantage in the application of NeabNPV. Aerial pesticide applications consisting of small spray droplets (<100 μm) generally provide greater efficacy in forest environments but are more prone to drift than larger droplets [31]. The propeller and wings generate a wake that extends several winglengths behind an aircraft as it flies through the air [32]. Aircraft wakes typically sink at 0.3–1.0 m/s [32], pushing the smaller (40–80 μm) droplets downward into the forest canopy [33]. Following the dissipation of the aircraft wake, spray droplets are affected by wind, air temperature, humidity, and characteristics of the ground cover [34], and small droplets may remain in the air column for hours after application [35]. As a result, active ingredients can be found hundreds of meters from the point of application [36,37,38,39,40,41]. Droplet suspension and eventual dispersal likely occurred at Island Pond where there was little wind during the application period (Figure 2A) and detection of spray droplets on Kromekote cards only to 25 m outside the treatment block (Figure 3A). Persistent winds and wind gusts (Figure 2B) carried NeabNPV-containing spray droplets into the areas on either side of the spray swaths at Old Man’s Pond (Figure 5A).

### 2.3. Significance of NeabNPV Field Trials

Before 1989, balsam fir sawfly population outbreaks in western Newfoundland were localized within the region between Stephenville and Corner Brook and were short in duration (3–4 y) [18]. The population outbreak that began after 1989, however, expanded to Corner Brook and beyond [18], and between 1990 and 2010, resulted in a total of 574,633 ha of moderate to severe defoliation in balsam fir forests, mostly in western Newfoundland [28]. A contributing factor to this unprecedented spread has been the extensive practice of precommercial thinning of regenerating balsam fir stands in the region [18,42]. Precommercial thinning, where the number of young stems is reduced in order to concentrate growth on the remaining trees, has been shown to affect the overall survivorship of balsam fir sawflies, resulting in population oscillations with higher amplitudes compared with populations in unthinned stands [42]. A lack of acceptable control options led the Canadian Forest Service and its partners to research and develop NeabNPV as means to suppress balsam fir sawfly populations [26,43]. The commercial product Abietiv™, with NeabNPV as the active ingredient, was registered with the Pest Management Regulatory Agency of Health Canada in 2006 [26] and 2009 [44]. The field trials carried out in Newfoundland in 2000–2005 demonstrated NeabNPV efficacy and were a required and essential component of the registration dossier [26]. In addition to the 22,500 ha of balsam fir forest treated with NeabNPV during the field efficacy trials [26], the Newfoundland and Labrador Department of Natural Resources treated an additional 40,000 ha with Abietiv™ in 2006–2009 [29].

## 3. Materials and Methods

### 3.1. Aerial Application of NeabNPV

NeabNPV occlusion bodies (OBs) were applied to stands of balsam fir using two Cessna 188B AgTruck aircraft (C-FIMY and C-GWKT; Forest Protection Limited, Lincoln, NB, Canada) [27]. The aircraft were equipped with MicronAir (Bromyard, Herts, UK) AU4000 rotary atomizers and AG-NAV (Newmarket, ON, Canada) aerial management systems [45]. NeabNPV was applied in a 20% aqueous solution of molasses at ultra-low volumes of approximately 2.5 L/ha. Aircraft speed during applications was 177 km/h with a track flow rate of approximately 20 L/min. The AU400 rotary atomizers were set at 6,000 rpm to generate droplets approximately 80 μm in diameter [17]. Spray swaths for each aircraft were approximately 25 m wide, and the aircraft flew adjacent, parallel spray lines, separated by 25 m, resulting in complete coverage by the aerosol spray at ground level. For experimental applications, sufficient NeabNPV OBs were added to the aircraft hoppers to result in an application rate of 1 × 10^9^ OBs/ha when applied to a full hectare [27].

### 3.2. Amplification and Purification of NeabNPV for Use in Field Trials

Stocks of NeabNPV OBs were produced as described previously [17,27]. Briefly, NeabNPV was applied at a rate of 3 × 10^9^ OBs/ha over balsam fir stands infested with high density populations of second- and third-instar balsam fir sawfly larvae. Approximately 7 d following NeabNPV applications and for the next 5–7 d, NeabNPV-infected larvae were collected onto plastic tarpaulins placed under individual trees by beating mid to lower canopy branches with plastic leaf rakes. Larvae and needles were then transferred to 20- and 40-kg woven polypropylene bags (normally used for sugar) along with a few balsam fir boughs for any larvae that were still feeding. The bags were stored upright and indoors at ambient temperatures of 15–20 °C for 7–10 d or until all feeding activity had ceased. Dead larvae, needles, and other debris were then transferred to 20-kg brown paper bags, which were stapled shut and stored at ambient temperature (approximately 20 °C). Dead larvae were separated from needles and other debris using a blower [27]. Larvae were then hand-picked from the remaining debris, placed in 50-mL conical, plastic centrifuge tubes and stored at −20 °C. NeabNPV OBs were isolated by first thawing and then rehydrating these larvae in 0.3% sodium dodecyl sulphate (SDS). Larvae were homogenized using a hand-held blender, and the OBs isolated using a combination of filtration and centrifugation [17]. The concentration of OBs was quantified by proportional counting using serial dilutions of a suspension of latex beads [17]. The mean concentration of OBs was adjusted to 4 × 10^9^ OBs/mL and then stored at 4 °C to inhibit the growth of contaminating bacteria.

### 3.3. Field Sites and Study Design

Field trials and assessments were conducted in July and August 2002 near Corner Brook, NL, Canada (48°57'N:57°57'W) in naturally regenerated and precommercially thinned stands of mixed conifers, dominated by balsam fir, but also including some black spruce (*Picea mariana* (Mill.) B.S.P.) and white spruce [*P. glauca* (Moench.) Voss] [46]. Two experimental blocks, each with its own untreated control block at least 1 km away, were set up (Figure 1). The purpose of the first block, near Island Pond [17] (block 02-T3: 48°53'13.0''N:57°52'55.9''W; control 48°53'48.5''N:57°53'17.7''W), was to examine the spread of NeabNPV from a 313-ha aerial treatment block into populations of balsam fir sawflies adjacent to the treated block. The purpose of the second 2,093-ha treatment block, near Old Man’s Pond [17] (block 02-T1: 49°3'23.3''N:57°51'45.6''W; control 49°05'37.3''N:57°54'49.0''W), was to determine whether or not NeabNPV could disperse and infect balsam fir sawfly larvae within a 200-m zone between spray lines. Temperature, wind speed, and relative humidity were monitored on site every 60 s at each treatment block during aerial applications of NeabNPV using a WatchDog (Plainfield, IL, USA) model 700 weather station.

The Island Pond treatment block (Figure 1) was sprayed on 22 July 2002 between 06:30 and 07:40 h [17]. The entire 313-ha block was treated with NeabNPV. The eastern half of the Old Man’s Pond treatment block, which contained our transect line (Figure 1), was treated on 24 July 2002 between 19:50 and 20:40 h [17]. The western half was treated on 25 July 2002 between 07:35 and 08:25 h [17]. NeabNPV was applied to both blocks at track flow rates of 17.5–20 L/min, with swath widths of 25 m. The Old Man’s Pond block was sprayed in treatment zones, 200 m wide, which were separated from each other by a 200 m wide area of untreated forest (no treatment zones). Spray deposit in both the Island Pond and Old Man’s Pond treatment blocks was monitored using 10 × 10 cm Kromekote cards (Smart Papers, Hamilton, OH, USA) placed on top of 1-m wooden stakes driven into the ground adjacent to branch sample sites located at 20-m intervals along transects roughly perpendicular to the spray tracks of the aircraft [17] (Figure 1). Kromekote cards were collected between 1 and 2 h after the spray at Island Pond, and within 1 h of the spray at Old Man’s Pond. 

### 3.4. Balsam Fir Sawfly Sampling

At Island Pond, one balsam fir tree was selected along each of two transects at distances of 0 (the edge of the treatment block), 25, 50, 100, 200 and 400 m outside of the treatment block (*i.e.*, total of six trees per transect). The two transect lines were established on opposite sides of the treatment block running roughly parallel to the direction of the prevailing north-east winds (Figure 1) in order to account for any drift that may occur from the spray. Transects at Island Pond were sampled on a weekly basis, starting with a pre-spray sample on 21 July and followed by post-spray samples on 1, 8 and 14 August. Two similar transect lines were established, and sampled weekly in a similar manner, in the control block. At Old Man’s Pond, a 400-m long transect line that ran perpendicular to the aircraft spray lines was established. The transect line spanned one untreated zone and extended 100 m into the two treatment zones on either side (Figure 1). A similar 400-m transect line was established, and sampled weekly, in the control block (Figure 1). One balsam fir tree was selected for sampling every 20 m along the transect line in both the treatment and control blocks (*i.e.*, a total of 20 trees per transect). Transects at Old Man’s Pond were sampled on a weekly basis, starting with a pre-spray sample on 22 July, and followed by post-spray samples on 29 July, 6 and 13 August in 2002 and on 2 July and 13 August 2003. Densities of balsam fir sawfly larvae and/or pupae (juvenile balsam fir sawflies) were determined by cutting a 45-cm branch tip, using pruning shears, from one mid-crown branch of each sample tree within each block pair (*i.e.*, treatment and control) on the same day. Sample branches were placed into 20-kg brown paper bags for transport to the laboratory at the Canadian Forest Service Field Station at Pasadena, NL (49°01'29.9''N:57°35'24.1''W) for processing. The numbers of larvae and cocoons on each sample branch were counted and recorded. To express balsam fir sawfly densities in terms of the number of juvenile balsam fir sawflies per m^2^ of foliage, the surface area of each branch was estimated by multiplying the 45-cm branch length by the average width of each individual branch. To determine the prevalence of NeabNPV, a sub-sample of 10 juvenile balsam fir sawflies (either living or dead) was randomly taken from each sample branch on each sampling date. Insects were individually placed into 1.5-mL polypropylene microcentrifuge tubes (Fisher Scientific, Fair Lawn, NJ, USA) and stored at −20° for molecular probing.

### 3.5. Molecular Probing for NeabNPV

Larvae and pupae were probed for NeabNPV using NeabNPV DNA-fluoroscein-N6-dATP-labeled DNA probes (Renaissance, Perkin-Elmer Life Sciences, Waltham, MA, USA). Seven NeabNPV DNA/*Eco*R1 fragments (3.5–5.5 kb) were used as templates [17]. Individual insects were thawed and homogenized in ~1 mL of double-distilled water in the 1.5-mL microcentrifuge tubes they had been stored in. A 3-µL aliquot of each sample was blotted onto Biodyne A nylon membranes (Pall, Gelman Laboratory, Port Washington, NY, USA). Positive controls of purified NeabNPV DNA or NeabNPV OBs were also spotted onto each membrane. Membranes were soaked in denaturing solution (0.5 N NaOH, 1.5 M NaCl) and incubated at 65 °C for 30 min. Membranes were neutralized in 1.5 M NaCl, 0.5 M Tris. pH 7.0 for 1 min, soaked for 5 min in 10× SSC (saline sodium citrate buffer), air dried on filter paper, and target DNA was then bound to the membranes by exposure to 125 mJ of UV radiation using a BioRad (Hercules, CA, USA) GS Gene Linker™. Membranes were soaked in hybridizing solution containing the labeled probe for 18 h at 65 °C. Excess probe and probe bound to non-specific DNA were removed with high stringency washes and results were recorded on BioMax ML film (Kodak, Rochester, NY, USA). The lower detection limit for the probing protocol was 5 × 10^3^ OBs [17], implying a positive detection only for specimens where NeabNPV had replicated. 

### 3.6. Statistical Analysis

Data from the two sites were analyzed separately because the NeabNPV treatments at Island Pond (entire block treated with sampling mostly outside the treatment block) differed from that at Old Man’s Pond (partial block treatment with sampling within the block). The independent and interacting effects of NeabNPV application (treatment) *vs.* no treatment (control), time of sample, and distance from NeabNPV application (a covariate with values of 0 to 400 m at Island Pond and 0 to 100 m at Old Man’s Pond) on juvenile balsam fir sawfly densities and percentage NeabNPV infection were evaluated using analysis of covariance in the General Linear Model in ANOVA (Minitab^®^, State College, PA, USA). Pearson’s correlation coefficients were calculated to evaluate the relationships between distance from spray swaths and the level of NeabNPV infection and the larval density in the treatment blocks.

## 4. Conclusions

Occlusion bodies of NeabNPV can only be produced in living larvae of its host, the balsam fir sawfly [17,30]. Currently, the only economical and effective way to do this for large-scale operational control programs against this forest insect pest is by production in high-density field populations of balsam fir sawfly larvae [17,27]. Reliance on field production can potentially limit supplies of NeabNPV OBs and, as a consequence, the registered biological control product, Abietiv™ [44]. In the present study and previously [17], it has been demonstrated that aerial application rates as low as 1 × 10^9^ NeabNPV OBs/ha can be sufficient to prematurely collapse increasing and peaking populations of balsam fir sawflies. Efficacious applications of NeabNPV OBs at these low rates depend on sufficient balsam fir sawfly larval population densities where a few individuals can be infected and then efficiently transmit NeabNPV horizontally to other cohort members [30,47,48]. Additionally here, we have demonstrated how aerial application strategies, such as the one we used at Old Man’s Pond employing aerosol drift and horizontal transmission of NeabNPV, can be used effectively for gammabaculovirus-based biological control products like Abietiv™ that may be expensive and/or in limited supply.
